# Performance of Topology Tests under Extreme Selection Bias

**DOI:** 10.1093/molbev/msad280

**Published:** 2023-12-24

**Authors:** Etai Markowski, Edward Susko

**Affiliations:** Department of Mathematics and Statistics, Dalhousie University, Halifax, NS, Canada; Apple Incorporated, Cupertino, CA, USA; Department of Mathematics and Statistics, Dalhousie University, Halifax, NS, Canada

**Keywords:** topology tests, phylogenetics, maximum likelihood, KH test, SH test, AU test

## Abstract

Tree tests like the Kishino–Hasegawa (KH) test and chi-square test suffer a selection bias that tests like the Shimodaira–Hasegawa (SH) test and approximately unbiased test were intended to correct. We investigate tree-testing performance in the presence of severe selection bias. The SH test is found to be very conservative and, surprisingly, its uncorrected analog, the KH test has low Type I error even in the presence of extreme selection bias, leading to a recommendation that the SH test be abandoned. A chi-square test is found to usually behave well and but to require correction in extreme cases. We show how topology testing procedures can be used to get support values for splits and compare the likelihood-based support values to the approximate likelihood ratio test (aLRT) support values. We find that the aLRT support values are reasonable even in settings with severe selection bias that they were not designed for. We also show how they can be used to construct tests of topologies and, in doing so, point out a multiple comparisons issue that should be considered when looking at support values for splits.

## Introduction

We will consider the performance of several tests in the presence of extreme selection bias. Selection bias here is unrelated to natural selection and instead refers to biases that occur when one selects hypotheses or results to report based on the data being analyzed. A common selection bias in phylogenetics, one that is a focus of this article, is that, in testing a tree of interest, the alternative tree hypothesis is usually the estimated tree. The alternative hypothesis is “selected” based on the tree being considered a good fit for the data. The first 2 tests considered, the Kishino–Hasegawa (KH) (Hasegawa and Kishino 1989; Kishino and Hasegawa 1989) and chi-square tests (Susko 2014), are not designed to deal with selection bias and so the question of interest is the effect that selection bias has on these tests. The other tests, the approximately unbiased (AU) (Shimodaira 2002) and Shimodaira–Hasegawa (SH) tests (Shimodaira and Hasegawa 1999), are designed to deal with selection bias. We add to this list another test, which we refer to as the Bonferroni test, that can be used to correct any 2-tree test. We use it here to correct the chi-square test; the Bonferroni test was previously described in Muñoz-Gómez et al. (2022) but its properties will be developed in more detail here.

Selection bias (Shimodaira and Hasegawa 1999; Goldman et al. 2000) leads to the expectation that 2-tree tests, like the KH and chi-square tests, will have elevated Type I error rates when applied in settings where the 2 trees are not fixed in advance. For 2-tree tests, there is a tree under the null hypothesis (Tree *H*) and a fixed tree for comparison under the alternative hypothesis (Tree *A*). The theory for these tests is intended to approximate the distribution of the differences in log likelihoods between Tree *A* and Tree *H* when some version of Tree *H* is correct. If the calculated difference for the data at hand is large relative to what is expected from this distribution, Tree *H* is rejected. In practice, however, it is usually inference only about a Tree *H* that is of interest. Can it be rejected or not? Because a natural fixed Tree *A* is not available, the maximum likelihood (ML) tree is used in its place. The reason that using the ML tree is expected to bias 2-tree tests is that, by definition of being an ML tree, it always has at least as large a log likelihood as one would have obtained for any particular fixed tree. Consequently, the distribution of the difference in log likelihoods between ML tree and Tree *H* tends to be larger than differences that one would have obtained comparing a fixed Tree *A* against Tree *H*, which is the distribution 2-tree tests are designed to approximate. Thus using a reference distribution for 2-tree tests gives thresholds for rejection that are too small. Type I error rates are expected to be inflated if they are used with the ML tree.

We consider simulations from 6 and 8 taxon trees where, for many of the settings, some subset of the edge-lengths are 0. These settings are chosen because selection bias is expected to be more extreme than in other cases. In the 6 taxon case, for instance, the maximized log likelihood is the maximum of the 105 log likelihoods corresponding to each of the possible trees. If some splits in the true tree have substantial edge-lengths, however, topologies that do not have these splits present will have small likelihood because there will be signal in the data for these splits. For instance, if the true tree splits taxa 1,…,6 as 12|34|56, and the edge-lengths leading to 34 and 56 are substantial but the edge-length leading to 12 is not, log likelihoods will usually only be large for trees that split 34 (and 56) from the other taxa. This leads to the maximized log likelihood being, in effect, a maximum over just 3 trees that have these splits; the remaining split that defines the tree is either 12|3456, 134|256, or 156|234. By contrast, if the generating tree were the star tree, every tree has a reasonable chance of giving the largest log likelihood.

We also illustrate how tests for trees can be used to calculate support values for splits in the ML tree. We compare the performance of the support values coming from the chi-square test, with and without Bonferroni correction, to the comparable likelihood-based aLRT values of Anisimova and Gascuel (2006). Somewhat surprisingly, the aLRT values are found to perform well, even in the presence of extreme selection bias; they were not designed to deal with such biases. Just as tests of trees can be used to calculate support values, support values can be used to construct test of trees. We illustrate how this can be done, point out that multiple comparisons corrections are needed, and investigate performance under the same extreme selection bias settings.

## Results

### An Overview of Testing Methods

We begin by considering an overview of the testing methods. More detailed information is available in the Materials and Methods section.

Topology tests have a null hypothesis that a particular topology, *τ*, gives the true tree. When selection bias is not present, tests are often designed to be conservative rather than accurate; the probability that an *α*-level test rejects the true tree is less than *α* rather than approximately equal to *α*. Tests can equivalently be considered as a procedure for getting a (1−α)×100% confidence set of trees, which is largely how they will be viewed here. The confidence set is the set of trees that give a *P*-value larger than *α*.

The existing tests considered here are the KH, SH, chi-square, and AU tests. The KH, SH, and chi-square tests all use as a test statistic the difference in log likelihoods between the ML tree, $\hat{\tau }$ and *τ*. The AU test differs in using a type of bootstrap support for *τ*. The KH and chi-square tests are tests of 2 trees that do not adjust for selection bias whereas the AU and SH tests do.

We also consider an approximate Bonferroni correction to the procedure that rejects whenever a 2-tree test of $H_{0}\;:\;\tau$ against $H_{A}\;:\;\tau^{\prime }$, rejects for a $\tau^{\prime }$ that is compatible with the strict consensus tree, $\tau_{0},$ of *τ* and $\hat{\tau }$; correction is needed because there is a multiplicity of tests. The correction is usually applied to the chi-square test (which we refer to as the Bonferroni test) here but could be applied to any 2-tree test. To ease computational cost, the default approach here applies the Bonferroni correction to the *P*-value determined from the test of *τ* against $\hat{\tau }$ which makes it more conservative than direct Bonferroni correction which would use the minimum *P*-value over $\tau^{\prime }$ in its place.

Another way in which topological uncertainty is assessed is through support for splits in a tree. A split corresponds to an edge in a tree and partitions the taxa into 2 groups A|B. A desirable statistical interpretation for split support is that it is (1−p)×100% where *p* is a *P*-value for the null hypothesis that the split is not present against the alternative hypothesis that it is well-resolved (Felsenstein and Kishino 1993; Anisimova and Gascuel 2006). With this interpretation, the hypothesis that a split is present can be accepted at the 0.05 level if its support is at least as large as 95%. The aLRT split support value is intended for splits in the ML tree and applies a Bonferroni correction (different than the one above) to the likelihood ratio test that the edge-length associated with a split is 0. The Bonferroni correction is intended to correct for the tree being the ML tree rather than fixed a priori.

We argue that topology tests can be used to give conservative split support values. The support value is calculated as (1−p)×100% where *p* is the largest *P*-value among trees that did not have the split. Similarly, we argue that split supports can be used to construct a topology test by including a tree in the (1−α)×100% confidence set if each of its splits have support at least (1−α). Because of the multiplicity of splits considered, however, we argue that a correction is needed. A Bonferroni correction applied to the aLRT test gives support (1−(m−3)(1−aLRT))×100% to a split, where *m* is the number of taxa; we refer to such values as aLRTc values.

### Real Data Illustrations

To illustrate the testing procedures with real data we first consider the HIV data set that Goldman et al. (2000) considered in a testing context. The data consist of a set of 6 homologous sequences, each 2000 base pairs long, from the gag and *pol* genes for isolates of HIV-1 subtypes A, B, D, and E: A1(Q23), A2(U455), B(BRU), D(NDK), E1(90CF11697), and E2(93TH057). The model fit to get log likelihoods was the GTR+G model. Table 1 gives the support values for the ML tree, which can be described in terms of its splits, B,D|A1,A2,E1,E2, E1,E2|A1,A2,B,D and A1,B,D|A2,E1,E2. The split support for the first of these splits was 100% for all tests and E1,E2|A1,A2,B,D was supported by all but the SH test. There was uncertainty about the A1,B,D|A2,E1,E2 splits, which were not supported by the SH, KH, and AU tests but were supported by the rest. For this data set, the support for splits in the ML tree largely explain the uncertainty in tree estimation. Table 2 gives results for the trees giving the top 4 log likelihoods. Consistent with their split support, the chi-square, aLRT-based test and their corresponding conservative analogs, Bonferroni and aLRTc, all support only the ML tree. The AU and KH tests both give the same 3 trees in their 95% confidence sets, the ones corresponding to nearest neighbor interchanges of the split A1,B,D|A2,E1,E2; both trees give similar log likelihoods. The SH test included a total of 15 trees in its confidence set. The only inference that could be drawn from it was that there was significant evidence for the B,D|A1,A2,E1,E2 split.

**Table 1:** ML tree split support for the HIV and Mammalian Mitochondrial data sets

	SH	KH	AU	$\chi^{2}$	Bo	aLRT	aLRTc
HIV data							
B,D|A1,A2,E1,E2 :	100.0	100.0	100.0	100.0	100.0	100.0	100.0
E1,E2|A1,A2,B,D :	60.2	98.8	99.5	100.0	100.0	100.0	100.0
A1,B,D|A2,E1,E2 :	13.1	80.0	73.8	99.5	98.5	99.3	97.8
Mammalian mitochondrial data							
Sl,Cw|Hm,Rb,Ms,Op :	95.7	99.9	100.0	100.0	100.0	100.0	100.0
Ms,Op|Hm,Sl,Cw,Rb :	37.8	90.5	87.9	100.0	100.0	100.0	100.0
Hm,Sl,Cw|Rb,Ms,Op :	3.5	52.7	36.9	72.5	38.2	64.2	− 7.4

**Table 2:** *P*-values for the trees with the largest log likelihoods among all trees for the HIV and Mammalian Mitochondrial data sets

dLnL	SH	KH	AU	$\chi^{2}$	Bo	aLRT	aLRTc	Topology
HIV data								
0.000	1.000	0.800	0.837	1.000	1.000	1.000	3.000	(A1,(A2,(E2,E1)),(B,D)) ;
3.964	0.869	0.200	0.262	0.005	0.015	0.007	0.022	(A1,(E2,E1),(A2,(B,D))) ;
4.266	0.867	0.177	0.258	0.003	0.010	0.007	0.022	(B,((A2,A1),(E2,E1)),D) ;
21.621	0.398	0.012	0.005	0.000	0.000	0.000	0.000	(B,((A2,A1),(E2,E1)),D) ;
Mammalian mitochondrial data								
0.000	1.000	0.527	0.674	1.000	1.000	1.000	3.000	(Hm,(Sl,Cw),(Rb,(Op,Ms)));
0.597	0.965	0.473	0.631	0.275	0.618	0.358	1.074	(Op,(Hm,(Rb,(Sl,Cw))),Ms);
8.694	0.841	0.103	0.083	0.000	0.000	0.358	1.074	(Op,((Rb,Hm),(Sl,Cw)),Ms);
18.020	0.622	0.095	0.052	0.000	0.000	0.000	0.000	((Op,Hm),(Rb,(Sl,Cw)),Ms);
20.476	0.562	0.067	0.121	0.000	0.000	0.000	0.000	(Op,Hm,((Rb,Ms),(Sl,Cw)));
20.957	0.547	0.028	0.047	0.000	0.000	0.000	0.000	(Sl,Cw,(Op,(Hm,(Rb,Ms))));
21.068	0.547	0.058	0.013	0.000	0.000	0.000	0.000	(Op,(Rb,(Sl,Cw)),(Hm,Ms));

The mammalian mitochondrial data were considered in Goldman et al. (2000) and Shimodaira (2002). It was also considered in Susko (2014), but in a context where only 2 trees were considered, whereas here the confidence sets come from analyses of all 105 possible 6-taxon trees. Table 1 gives the support values for the ML tree fit to the 3414-site alignment using a mtRev+F+G model. The labeling here is


$$\begin{array}{rl} Hm & =human,\;Sl=seal,\;Cw=cow,\;Rb=rabbit,\\ Op & =opossum,\;Ms=mouse. \end{array}$$

There is little uncertainty that *seal* and *cow* should be split from the rest. For the SH, KH, and AU tests, there is some uncertainty that *mouse* and *opposum* should be split from the rest although support values are larger. The chi-square, aLRT-based test and their conservative analogs, by contrast, infer this split to be strong supported. The situation differs for the split Hm,Sl,Cw|Rb,Ms,Op, which is not well supported by any test. The aLRTc value is negative. This is because it is not truly a support value but rather a conservative multiple comparisons correction to the aLRT. By contrast with the HIV example, tree testing results (Table 2) are a refinement of the inferences that one can draw from split support. When one split, Hm,Sl,Cw|Rb,Ms,Op, is not supported, that suggests there will be 3 trees in its confidence set. For aLRT and aLRTc, this is the case, as it must be since the corresponding tests are derived solely from its support values. But the 95% chi-square and Bonferroni confidence sets only include 2 trees because the other tree has a substantially larger log likelihood. The AU and KH test include 6 trees. Two of the trees in the AU confidence set are not among those listed. Because the AU test does not use the likelihood ratio as a test statistic directly, it can happen that some of the trees excluded from its set have larger log likelihoods than some of the trees that are included in its set. Interestingly, the KH test does not include the sixth ranked tree in terms of log likelihood but does include the seventh. The KH test uses the log likelihood ratio as a test statistic but also uses the variance of the site log likelihood differences (ML tree vs tested tree). Consequently, a tree with a smaller log likelihood ratio can give a larger *P*-value if its variance is larger. The SH 95% confidence set includes all 15 trees with *seal* and em cow split from the rest.

**Fig. 1.**
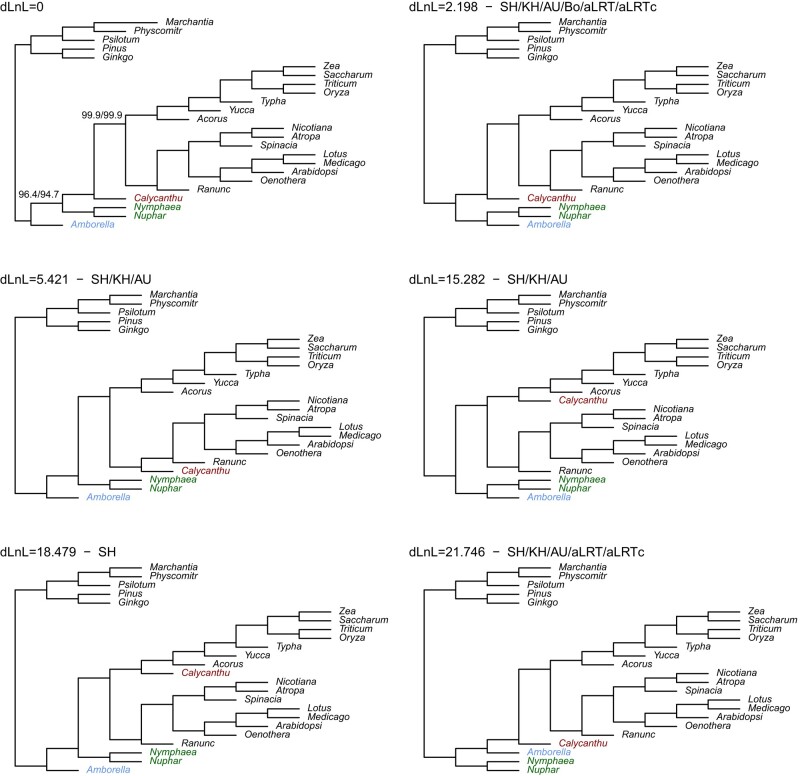
The topologies giving the largest log likelihoods for the Amborella data and the methods that include them in their 95% confidence set. Support values less than 100% are indicated on the ML tree for $\chi^{2}$/aLRT.

The final illustrative set considered will be labeled the Amborella data set (Leebens-Mack et al. 2005), because the location of *Amborella* is one of the sources of uncertainty. This is illustrated in Fig. 1. The only chi-square or aLRT support values that differ substantially from 100% are those associated with the split that places *Amborella* at the base of the angiosperms. The next best tree, in terms of log likelihood, has Amborella grouping with the water lillies. Only the chi-square test does not include it in its 95% confidence set, and then only marginally, as the support value of 96.4% is close to 95%. The alignment here consists of 24 taxa and has 15,688 amino acid sites. The model fit was the JTT+F+G model. The aLRT and aLRTc confidence sets always include all tree topologies associated with an unsupported split and so it also includes the splits with the sixth largest log likelihood, in spite of this involving a rather large increase in log likelihoods. Because this is a large data set, a candidate set for confidence set inclusion cannot include all possible trees. The set here was constructed as the set of bootstrap trees over 100 bootstrap samples plus all trees that differ by one nearest neighbor interchange from the ML tree. The confidence sets for the SH, KH, and AU tests included 52, 6, and 7 trees, respectively. The alRT/aLRTc sets had 3 trees whereas the chi-square test gave 1 tree and its Bonferroni correction picked up an additional tree.

### Simulation Results

**Fig. 2.**
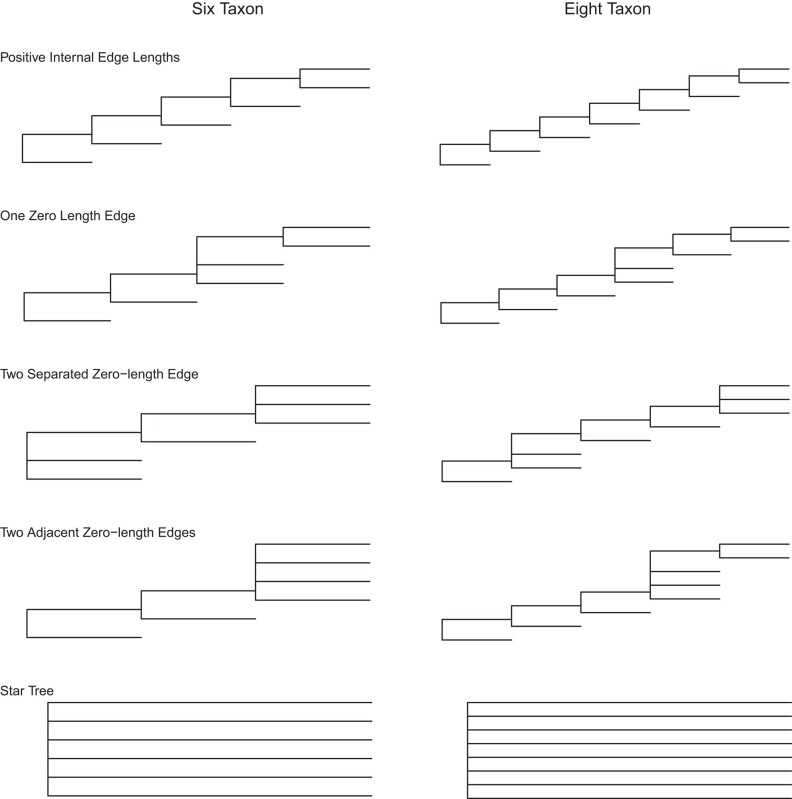
Trees used in simulation.

We consider 2 sets of simulations. One with 6 taxa and another with 8. For a fixed set of taxa, a number of tree settings were considered that can be described in terms of the edge-lengths that were set to zero and whether those zero-length edges were adjacent or not. For both 6 and 8 taxon settings, we also simulated from a star tree. The trees are illustrated in Fig. 2. To push the boundaries of selection bias, we consider trees that not only have edge lengths that are close to 0 but also have some edge-lengths that are exactly 0. Selection bias is expected to get worse when the set of trees competing for ML status is large. If the tree is well-resolved, because of the signal that will be present in the data for true splits, trees without those splits will have small likelihood. The set of trees competing for ML status is thus effectively small. By contrast, with zero edge-lengths, even if there are a relatively large number of sites, sets of trees compatible with the true tree will always be in competition for ML status.

For any fixed tree setting, we generated data sets with sequence length 1,000. The substitution model was the HKY model with κ=2 and frequencies of nucleotides $\pi_{A}=0.1$, $\pi_{C}=0.2$, $\pi_{G}=0.3$, and $\pi_{T}=0.4$. Simulations included rates-across-sites variation from a 4-category gamma rates-across-sites model with α=1. We generated 1,000 data sets for each 6-taxon setting and 100 data sets for each 8-taxon setting. Internal edge-lengths varied across settings but terminal edge-lengths were always set to 0.1.

The main statistical properties considered are coverage and mean confidence set size. Mean set size is the average number of trees in the confidence set for a procedure, averaged over simulated data sets. Coverage is the proportion of simulated data sets that had the true tree contained in the confidence set. Because we consider trees that have some edge-lengths that are exactly 0, multiple trees are true. Coverage in such cases is calculated as the average coverage over all of the true trees.

We start our discussion of simulation results with a brief presentation of a problem with AU tests that caused us to consider a slight modification of the default IQ-TREE implementation in all further results; the modified version was used in the previous real data illustrations. In the Materials and Methods section of this article, we review the testing procedures and explain why the AU *P*-value construction method can become unstable when bootstrap support for a topology is very low; bootstrap support is alternatively referred to as bootstrap probability or BP. Since IQ-TREE reports BP as well as the AU *P*-value and since BP = 0 suggests little evidence for the tree, a simple correction is to set the AU *P*-value to 0 whenever BP = 0. That uncorrected AU can give anomalously large *P*-values in such cases is well-illustrated by considering a few results with and without correction. For simulation from a 6-taxon tree with well resolved internal edge-lengths ($t_{I}=0.1$) the correction did not matter but when the tree was more poorly resolved $\left(t_{I}=0.001\right)$ the mean confidence set size without correction was approximately 91 whereas it was 78 with correction. Thus, on average, for a given data set, 13 trees that had BP = 0 were included in the uncorrected AU confidence set. With 8 taxa the problems became even more pronounced. Even for a well-resolved tree with $t_{I}=0.1$, the mean set size of the uncorrected AU confidence set was 11 whereas it was approximately 1 after correction. For a more poorly resolved tree with $t_{I}=0.01,$ the average confidence set sizes were 808 and 160. We consider the corrected version of the AU test in all further discussion and recommend that it be applied generally when reporting results of the AU.

Results for all tests in 6 taxon simulations are given in Table 3. When the internal edge-lengths, $t_{I}$, are 0.1, all of the methods perform well most of the time. With a single exception (the SH test when all of the internal edge-lengths are positive), the mean number of trees in the 95% confidence set match the correct number of trees up to the precision indicated. Note that the numbers of trees that are compatible with the true tree are 3, 9 and 15 for the settings with one zero-length edge, 2 separated zero-length edges and 2 adjacent zero-length edges respectively. Coverages tend to be large with the exception of the chi-square test which gives coverage at or near the stated level. Having coverage closer to the stated level is usually desirable but for this edge-length choice there is little cost in it being too large. None of the methods include unnecessary trees in their confidence sets.

**Table 3:** Results for the 6-taxon simulations. Entries are Coverage/Mean set size (SD)

$t_{I}$	SH	KH	AU	$\chi^{2}$	Bo
Positive internal edge lengths					
0.1	100/5 (0)	100/1 (1)	100/1 (0)	100/1 (0)	100/1 (0)
0.01	100/63 (35)	100/42 (37)	100/36 (20)	99/8 (13)	100/29 (37)
0.001	100/105 (5)	100/105 (1)	92/78 (11)	89/86 (33)	99/104 (6)
One zero-length edge					
0.1	100/3 (0)	100/3 (1)	100/3 (0)	97/3 (0)	99/3 (0)
0.01	100/93 (33)	100/81 (26)	100/58 (18)	95/32 (35)	99/77 (37)
0.001	100/105 (3)	100/105 (0)	83/79 (11)	88/88 (31)	100/104 (4)
Two separated zero-length edges					
0.1	100/9 (0)	100/9 (0)	100/9 (0)	95/9 (1)	99/9 (1)
0.01	100/89 (36)	100/83 (31)	100/58 (21)	94/30 (36)	99/65 (43)
0.001	100/105 (3)	100/105 (0)	89/78 (10)	87/88 (31)	100/104 (5)
Two adjacent zero-length edges					
0.1	100/15 (0)	100/15 (0)	98/15 (1)	93/14 (3)	99/15 (1)
0.01	100/102 (22)	100/97 (14)	97/69 (15)	91/57 (41)	98/95 (24)
0.001	100/105 (2)	100/105 (0)	84/79 (10)	86/89 (30)	99/104 (4)
Star tree					
	100/105 (2)	99/105 (1)	76/80 (10)	86/90 (29)	100/105 (4)

When $t_{I}=0.01$, the estimation problem becomes more difficult and there is more variability across the methods. The SH and KH tests are conservative (excessive coverage and numbers of trees), much more so than the other methods. Both have coverage 100% but at a cost of usually including many more trees than the other approaches. The chi-square test arguably performs the best. It gives the smallest confidence sets of trees while maintaining coverage close to or above the stated coverage in most cases; the exception is a coverage of 91% when there were 2 adjacent zero-length edges. The Bonferroni test provides a conservative cross-check on the chi-square test. It has coverage close to 100% but includes a substantial number of trees. The AU test includes more trees than the chi-square test but consistently gives the second-best mean confidence set size.

When $t_{I}=0.001$, the problem becomes very difficult. The conservative SH, KH, and Bonferroni procedures have large coverage but almost always include all 105 trees and are thus uninformative. The chi-square and AU tests have smaller confidence sets, but set sizes are still large. The cost for these 2 tests is that they now have substantially lower coverage than the stated 95% confidence sets. Interestingly, the AU test, which gave larger coverage and set sizes than the chi-square in the moderately difficult case where $t_{I}=0.01$, now gives smaller coverage and confidence set sizes.

When the true tree is the star tree, every tree is correct. The right confidence set is all 105 trees so any smaller size corresponds to mistakes. This is the most severe case of selection bias. Note that whereas the chi-square test is expected to have an elevated Type I error rate when there is selection bias, the AU test was intended to adjust for selection bias. Yet, it has low coverage, lower than the chi-square test and much lower than the KH test. That the AU test has lower than expected coverage may be explained in part by theoretical difficulties with its motivation. These are discussed in the Materials and Methods section. The other surprise is the very high coverage of the KH test. It was not designed to adjust for selection bias and, consequently, should be expected to have elevated Type I error. Yet it has very high coverage over all settings. Because its coverage is so large, there is no need to correct it in the way that the SH does. Finally, we note that the set sizes and coverages with the star tree are comparable to most simulations involving $t_{I}=0.001$, which highlights that edge-lengths in that range make the problem very difficult.

Table 4 gives the results for the 8-taxon simulations. Since having internal edge-lengths of 0.001 gave performance that was very similar to the star tree in the 6-taxon case, only $t_{I}=0.1$ and 0.01 were considered in 8-taxon simulations. With the exception of the SH test, all of the tests do very well in the case that internal edge-lengths are 0.1. Whether $t_{I}=0.1$ or 0.01, the KH test is always conservative, with high coverage albeit at a cost of large confidence sets, in spite of these being settings that should result in a substantial selection bias. We have not found a setting where the KH test has undercovered. Because the KH test is so conservative, there is little reason to consider the SH test.

**Table 4:** Results for the 8-taxon simulations. Entries are Coverage/Mean set size (SD)

$t_{I}$	SH	KH	AU	$\chi^{2}$	Bo
Positive internal edge lengths					
0.1	100/24 (7)	100/1 (0)	100/1 (0)	100/1 (0)	100/1 (0)
0.01	100/1276 (1779)	100/164 (263)	100/160 (129)	98/14 (22)	100/179 (604)
0.001	100/10395 (0)	95/9507 (2182)	49/1095 (182)	78/4578 (4079)	100/10355 (182)
One zero-length edge					
0.1	100/37 (51)	100/3 (0)	100/3 (0)	95/3 (1)	98/3 (0)
0.01	100/2905 (3128)	100/615 (1293)	100/297 (175)	96/47 (106)	98/1112 (2443)
Two separated zero-length edges					
0.1	100/121 (40)	100/9 (0)	100/9 (1)	97/9 (3)	100/9 (2)
0.01	100/1692 (2498)	100/424 (1132)	100/234 (164)	96/56 (118)	99/579 (1845)
Two adjacent zero-length edges					
0.1	100/76 (8)	100/15 (0)	99/15 (0)	91/14 (1)	98/15 (1)
0.01	100/5843 (4010)	100/2097 (2832)	91/512 (238)	89/254 (533)	100/4112 (4297)
Star tree					
	100/10395 (0)	99/10247 (782)	11/1109 (176)	71/7335 (3884)	100/10394 (5)

As in the 6-taxon case, $t_{I}=0.1$ is a relatively easy case. Most of the methods have very high coverage and do not include any trees beyond those that are strictly necessary. An exception arises for the chi-square test which has coverage 91% for the tree with 2 adjacent zero edges. The mean set size is 14 with standard deviation 1, so one tree out of the 15 correct trees is being missed on average.

With $t_{I}=0.01$, the situation becomes considerably more difficult. The SH, KH, and Bonferroni procedures have large coverage over all settings but the cost is that their confidence sets include far more trees. The chi-square confidence sets are the smallest but both the chi-square and AU confidence sets are much smaller than KH and SH. Coverage for AU and the chi-square test were usually acceptable but dipped below the 95% nominal level to around 90% in the case of 2 adjacent zero-length edges.

The star tree is the most challenging selection bias case. All trees are correct, so the only mistake that can be made is not to include some. In addition, at 10,395, the correct set size is very large. The KH, SH test, and Bonferroni-corrected chi-square test nevertheless have very high coverage. By contrast, the coverage of the chi-square and AU tests drop down to 71% and 11%. The drop in coverage for the AU test is particularly striking here. As noted in the 6-taxon case, the behavior of the tests for the star tree has implications for the behavior when the tree is well-resolved but with short edges. To illustrate this, we considered an additional simulation when all of the edge-lengths were positive but with common internal edge-length 0.001. The chi-square and AU tests undercover with percentages of 78% and 49%. The set sizes are large enough here that the main information they convey is that there is little certainty about the true tree. But, it is concerning that the coverages of the true tree are so small for the AU test.

One of the reasons that the Bonferroni test over-corrects is that it uses the *P*-value for a 2-tree test that has the ML tree as an alternative tree. That *P*-value is intended as an approximation to the minimum *P*-value over 2 tree tests where the alternatives are those trees that are consistent with $\tau_{0}$, the strict consensus of the tree of interest, τ, and $\hat{\tau }$. Table 5 gives results for the subset of 6-taxon settings that have an internal edge-length of 0.01 ($t_{I}=0.01$). The results for the chi-square test here use the minimum *P*-value without correction and we see that this results in a more substantial effect of selection bias. We also considered using the chi-square test with a low degree of freedom correction (see Materials and Methods section) that makes it less conservative. Both versions of the chi-square are affected in a substantial way by selection bias when multiple edges are 0 and Bonferroni corrects this bias.

**Table 5:** Results for 6-taxon simulations and an internal edge-length of 0.01 using a less conservative Bonferroni correction with multiple tests (Bom) and the Barry–Hartigan correction (BH) applied to the chi-square test ($\chi^{2}$), the chi-square test with a low degree of freedom correction ($\chi^{2}$ DF) and the KHns test (KHns) where these tests use minimum *P*-values. Entries are Coverage/Mean set size (SD)

	Zero	One	Two separate	Two adjacent
$\chi^{2}$	97/7 (12)	93/28 (32)	90/24 (30)	87/48 (38)
Bom	98/26 (35)	97/70 (40)	97/59 (43)	98/91 (26)
BH	98/23 (33)	97/64 (42)	97/52 (44)	98/86 (32)
$\chi^{2}$ DF	95/5 (10)	88/22 (27)	83/20 (26)	80/40 (33)
Bom	98/23 (33)	95/66 (40)	95/56 (42)	97/88 (27)
BH	98/20 (31)	95/61 (42)	95/49 (43)	96/84 (32)
KHns	95/4 (7)	85/15 (20)	81/14 (19)	72/27 (29)
Bom	98/18 (29)	95/59 (42)	95/49 (43)	96/85 (32)
BH	98/15 (26)	95/50 (42)	94/41 (42)	95/74 (39)

Although the chi-square tests are affected by selection bias and less conservative than most other tests, they are still conservative as 2 tree tests. To see whether correction could be effective for an accurate 2-tree test that will be more affected by selection bias, we applied Bonferroni to the KHns test of Susko (2014) for the 6-taxon cases with edge-length 0.01. The KHns test is heavily affected by selection bias and the Bonferroni is an adequate correction.

All of the Table 5 results calculated *P*-values over multiple tests rather than a single *P*-value associated with the alternative hypothesis that the tree is $\hat{\tau }$ which has been the main case considered. When the *P*-values for multiple tests are calculated, more sophisticated corrections, like the Benjamini–Hochberg correction (Benjamini and Hochberg 1995) can be applied. The Benjamini–Hochberg correction will always give a smaller *P*-value than Bonferroni. Thus, as Table 5 illustrates, it will always give a smaller set size. We see as well that it maintains good coverage properties even for the KHns test.

### Support Values

Table 6 gives results using confidence sets to calculate support values for splits in the ML tree and makes comparisons with the aLRT support values of Anisimova and Gascuel (2006). Results are for the same 8-taxon settings. Considering the branch support results in the table, the aLRT support values give the largest power over all settings. The power of the chi-square support values are comparable whereas power tends to be much smaller with Bonferroni correction. Power is defined as the proportion of times that a split in the ML tree was supported, among ML splits that were also correct. Support values for the SH, KH, and AU tests were also calculated but gave unsatisfactory results, each giving power less than 10% in all settings where the internal edge length $t_{I}=0.01$.

**Table 6:** Eight-taxon branch support and confidence set results

	Correct		Branch support ML tree			Confidence sets			
$t_{I}$	Y	N	$\chi^{2}$	Bo	aLRT	$\chi^{2}$	Bo	aLRT	aLRTc
Positive internal edge lengths									
0.1	500	0	100/NA	100/NA	100/NA	100/1	100/1	100/1	100/1
0.01	461	39	67/3	40/0	70/0	98/14	100/179	100/29	100/74
One zero-length edge									
0.1	400	100	100/7	100/3	100/4	95/3	98/3	97/3	99/3
0.01	362	138	60/3	23/1	73/6	96/47	98/1112	94/49	99/648
Two separated zero-length edges									
0.1	300	200	100/2	100/0	100/2	97/9	100/9	98/9	100/9
0.01	284	216	71/1	33/0	81/2	96/56	99/579	96/152	99/191
Two adjacent zero-length edges									
0.1	300	200	100/2	100/0	100/5	91/14	98/15	92/14	100/15
0.01	251	249	48/1	10/0	69/8	89/254	100/4112	84/490	97/1506
Star tree									
	0	500	NA/0	NA/0	NA/7	71/7335	100/10394	68/7055	96/10010

Correct gives the numbers of times that a split in the ML tree was correct over simulations and splits. Support results are Power/Type I error rate, the percentages of times that a split in the ML tree had support above 95%, among cases where it was correct (Power) and cases where it was incorrect (Type I error). Confidence set results are Coverage/Mean set size. The aLRTc adjusts aLRT for multiple tests.

The Type I error rate is the proportion of times that a split in the ML tree was supported, among ML splits that were incorrect. For the chi-square and Bonferroni approaches, the Type I error rate tends to be low or acceptable, as expected by theory; see the Materials and Methods section. The aLRT was designed to have a Type I error rate of at most 5% but the probabilities for this result were calculated assuming only one edge of zero-length and that the ML tree is highly likely one of 3 trees, those where all other splits in the tree are estimated correctly. Such approximations might be expected to work reasonably well when the tree is well resolved or there is one zero-length edge. We see low Type I error rates in those cases. When there are 2 separated zero-length edges, one might also expect the approximation to work reasonably well and we see that too. But for the cases with 2 adjacent zero-length edges or a star tree, an increase in Type I error rates is expected as more than 3 trees are competing for ML status; the aLRT value is derived as a correction for taking a maximum over 3 trees. What we see, however, is that although the type I error rate increases, it is still acceptable even in the case of a star tree. This may be due to the aLRT always making comparison between the ML tree and the best tree without the focal split but keeping all the other splits present. We expect the maximized log likelihood to be larger when the true is the star than when it has only one unresolved edge, but we also expect the log likelihood for the next best tree to increase too, so that the difference doesn’t shift as much from one scenario to the other.

Although the aLRT support values perform well, a confidence set constructed with them is comparable in terms of performance to that of the chi-square test albeit sometimes with a substantially increased average set size. Like the chi-square procedure the confidence sets undercover in cases of extreme selection bias.

Confidence sets for aLRT are obtained here by including a tree in the confidence set if all of the supported splits in the ML tree are present in it. As discussed in the Materials and Methods section, a multiple comparisons adjustment is needed here. That adjustment is to declare a split supported if its support is above 1−α/(m−3) instead of 1−α, where *m* is the number of taxa. Results for it are given in Table 6 under the aLRTc column. This approach increases coverage to acceptable levels even with extreme selection bias. The cost, however, is that the average set size increases. We see, however, that the set size is not nearly as large as the Bonferroni test.

## Discussion

We have shown that the KH and SH test are very conservative tests, even under extreme selection bias, giving large coverage but at a cost of often including a large numbers of trees in their confidence sets. The chi-square test is also often conservative in such settings but more informative, often being the only test giving confidence set sizes that are practical for detailed comparison of trees in the set. It does sometimes undercover when there are a lot of poorly resolved edges in the tree. The Bonferroni-corrected *P*-values provide a conservative test. The behavior of the AU test was usually reasonable but a little less predictable. In some cases, it had very large coverage and set sizes, but it also gave very low coverage in some 8-taxon cases.

Based upon the results here, we recommend that the SH test be abandoned. It was intended as a selection bias correction for the KH test but what we have found is that the KH test is so conservative that no correction is needed even in extreme selection bias settings. Indeed, the KH performance was so conservative that we believe it should usually be considered a less desirable approach than the simple chi-square test. The KH test may, however, still be a useful conservative cross-check. If it rejects all but a single tree, for instance, that would suggest very strong support for that tree. That the KH test could not be expected to give correct *P*-values for 2-tree tests was established in Susko (2014). Why, theoretically, it behaves so conservatively is not well understood and deserves additional attention.

We did not consider tests with a large number of taxa. This kept computational cost manageable and avoided confounding factors such as tree search algorithms failing to converge and candidate set selection. Results can be expected to have relevance to settings where there are many taxa but some portion of the tree is poorly resolved. In this case, the subgroups of taxa that are well separated from each other should behave roughly like taxa in the present setting. We expect, however, that the uncertainty about relative evolutionary relationships within groups, as well as the uncertainty about character states at their ancestral nodes, will lead to larger confidence set sizes.

We also did not consider tests with a large number of sites. Due to the increasing certainty that arises with large number of sites, one can expect the set sizes to decrease as the number of sites increases. The same cannot be said of coverage. For most of the tests, motivation is based on large sequence-length properties of the log likelihood differences. Approximations may get better and so coverage without selection bias may improve but coverage is not expected to steadily increase. This implies that conservative tests like the KH test may become valuable in such settings. Although the confidence set sizes here were found to often be too large to be useful, they can be expected to decrease as the number of sites increases and the coverage can be expected to remain high.

The AU and chi-square tests had poor coverage properties for the star tree. Although the star tree case is a worst case scenario, it was shown that similar properties arise in a resolved but poorly resolved tree. Because the set sizes tend to be very large in such settings, the main inference is that there is little certainty about the correct tree. Since no strong conclusions would be drawn about the true tree, the low coverage might not be as substantial a concern as in other settings.

The AU test was shown to sometimes give large *P*-values when bootstrap support for the tree was 0. As explained in the Materials and Methods section, this is not because there is evidence that the tree is plausible in spite of low bootstrap support. Rather it is a consequence of numerical instabilities. Our correction was to set the AU *P*-value to 0 whenever BP = 0. We recommend that, at the very least, this correction be made when reporting AU results. However, the problem can be expected to arise more generally when BP≈0 and also when BP≈1, so it is difficult to be sure what effect that might have on the test’s performance. IQ-TREE uses a least-squares approach to calculating the AU *P*-value. It is possible that the ML approach outlined in Shimodaira (2002) Appendix, Point 9 might help to ameliorate the problem but it will not eliminate it. Whenever BP = 0, it is possible that the AU *P*-value will not be well defined. For this reason, it will always be important to compare its value with those of other tests.

The Bonferroni correction developed here can be applied to any 2-tree test. The Bonferroni correction is a generic multiple comparisons adjustment. In the case that tests are independent, it is an accurate adjustment but in cases where tests are positively dependent, as might be expected here, it over-corrects. In addition, it was applied here to the chi-square test. Because the chi-square test is a conservative test, the Bonferroni correction can be expected to be an over-correction and it was. It over covered the true tree but in 6-taxon cases gave a set size that was comparable to the KH test and in 8-taxon case gave set sizes that were considerably larger. It thus was not supported as an improvement over the KH test in simulations. It has, however, a better theoretical justification as a selection-bias corrected test than KH and did perform better when used with the more accurate KHns 2-tree test. The use of the KHns 2-tree test also illustrated that selection bias corrections are needed with accurate tests.

An alternative approach to dealing with selection bias is provided by the Swofford–Olsen–Waddell–Hillis (SOWH) test of Swofford et al. (1996) that was considered in greater detail in Goldman et al. (2000). A parametric bootstrap is used. For each tree considered for inclusion in the confidence set, data sets are repeatedly simulated from that topology and fixed to their estimated values. For each of these bootstrap data sets, the difference in log likelihoods is calculated between the ML tree for that data set and the tree being considered for inclusion in the confidence set. A *P*-value is then the proportion of bootstrap data sets yielding a difference in log likelihoods larger than the observed difference for the original data. As discussed by Susko (2014) in the context of 2 tree tests, the *P*-values from the SOWH test can be very small because using estimated edge-lengths often gives a well-resolved tree to simulate bootstrap data sets. Well-resolved trees tend to give smaller differences than would be expected under a null hypothesis where the tree is only partially resolved. It is better to use the consensus tree between the ML tree and tree being considered for inclusion as has been considered the null hypothesis tree here. The SOWH test has not been widely used in practice because of its computational complexity. ML estimation has to be repeated over every bootstrap data set and the entire process needs to be repeated for every tree considered for inclusion in the confidence set. Some computational savings may be available when using consensus trees to create parametric bootstrap data sets, as results for these can be reused when 2 differing trees considered for inclusion share the same consensus tree with the ML tree. Nevertheless, the approach is infeasible with large numbers of taxa.

We showed how tests can generally be used to alternatively provide support for splits. Results compared the chi-square support values with those of aLRT. The aLRT was found to have larger power across all scenarios. This won’t hold generally. As shown in the Materials and Methods section, the support values of the chi-square tests will be larger than aLRT in 4-taxon cases. Thus the chi-square test will have larger power than the aLRT in such settings, albeit possibly at a cost of Type I error rates that exceed *α* when rejecting splits with support greater than (1−α)×100%. The reason for lower power in the 8 taxon case is that trees that differ from the ML tree by more than one split are tested and sometimes not rejected by the chi-square test. These are never considered by the aLRT procedure which only makes comparisons to trees that differ by one split.

Because the aLRT procedure only makes comparisons between the trees that differ by one split, it makes sense that it would have inflated Type I error rates when the true tree was the star tree. In that case all trees have positive probability of being the ML tree, whereas the aLRT assumes only 3 are possible. This did not turn out to be the case. With 8 taxa and thus 10,395 competing trees, the Type I error rate of the aLRT using the 95% threshold was only 7%. The explanation seems to be that although there is additional information available that a split is not well supported that can be gleaned by making comparison with trees that differ by more than one split (chi-square had an error rate of 0%), comparison with just those trees that differ by one split suffices.

Using aLRT as a confidence set construction procedure did not give as good results. With the exception of the star tree, its mean set size always matched or exceeded that of the chi-square sets, sometimes substantially so. Yet its coverage was usually lower. Often its coverage was good but in the cases with 2 adjacent zero-length edges or a star tree, its coverage values of 92%, 84%, and 68% were below the nominal 95% targets. This is because there is another multiple comparisons issue here, one that we likely often do not realize when looking at support values informally. We are prone in such cases to treat 95% support for all splits as evidence for the entire tree being significantly supported. The reality is that because we are making multiple inferences about multiple splits, the chance that at least one of these is in error is larger than the chance that we make an error for one particular split. The aLRTc provides a simple correction and could provide a template for reporting support for an ML tree based on its split support values alone.

## Materials and Methods

### Coverage and Confidence Sets

We describe results in terms of confidence sets but they could equivalently be described in terms of testing properties. To see the equivalence, we note that a (1−α)×100% confidence set of trees can always be constructed from a given test as the set of trees that one fails to reject at the *α*-level. In other words, the set of trees, among the candidate trees, that gave a *P*-value larger than *α*.

The candidate trees that we consider is the set of all 105 possible trees in the 6-taxon case and all 10,395 trees in the 8-taxon case. In practice, particularly with large numbers of taxa, this set would usually be considerably smaller, would be based on a prescreen, and might include the top trees found during tree searches or trees compatible with a consensus tree found by comparing trees considered plausible based on previous studies. The goal here is to see how serious the effects of selection bias will be when confidence sets are constructed as they should be in theory. Considering all trees also avoids any confounding effects that a prescreen might have.

The main properties of confidence sets that we consider are coverage and mean confidence set size. Coverage of a confidence set is defined as the probability that the true tree is contained in that set. It is directly related to the Type I error of the test: coverage is equal to 1−P(Type I Error). The other property of tests that is often considered is power: the probability that an incorrect tree will be rejected. This probability will vary over the incorrect trees considered. Average power, over trees, can be shown to be linearly related to mean set size and coverage,


$$\text{Mean set size}=N_{I}-N_{I}\cdot \text{Average power}+N_{C}\cdot \text{Coverage},$$

where $N_{C}$ and $N_{I}$ denote the numbers of correct and incorrect trees in the candidate set. Thus power and mean set size are equally informative. We choose to report mean set size because it better illustrates the practical implications of increased power. Increased power gives smaller set sizes implying more conclusive statements about the sets of trees that might be plausible.

Finally, because we consider trees that have some edge-lengths that are exactly 0, multiple trees are correct. With one zero-length edge, 3 trees are correct, with 2 separated zero-length edges, 9 are correct and with 2 adjacent, 15 are correct. Consequently, the ideal confidence set sizes in these cases are those with all correct trees and should be 3, 9, and 15. Coverage in such cases is calculated as the average coverage over all of the true trees.

### A Review of Testing Procedures

The KH, chi-square, and SH tests all use as a test statistic the difference in log likelihood between the ML tree, $\hat{\tau },$ and the tree considered for inclusion in a confidence set, *τ*. These tests differ only in the distributions that they use to calculate a *P*-value. The AU test differs substantially from the other approaches in using a *P*-value calculated from bootstrap support for *τ* rather than a log likelihood ratio. All tests include *τ* in a (1−α)×100% confidence set if its *P*-value exceeds *α*.

#### The KH Test

The original KH test in Kishino and Hasegawa (1989) was a test of one tree, *τ* (null hypothesis), against another $\tau^{\prime }$ (alternative hypothesis). It applies a *z*-test to the site log likelihood differences for *τ* and $\tau^{\prime }$. Let p(x;θ,τ) and $p(x;\theta ,\tau^{\prime })$ denote the probabilities of site pattern *x* (e.g. x=AARR for 4 taxa) for trees *τ* and $\tau^{\prime }$, calculated when the parameters for the model are *θ*; parameters include edge-lengths and possibly other substitution parameters. Let $\hat{\theta }(\tau )$ and $\hat{\theta }(\tau^{\prime })$ denote the ML estimates of parameters for *τ* and $\tau^{\prime }$. Then, the differences used by the KH test are $d_{i}=\log\;p(x_{i};\hat{\theta }(\tau^{\prime }),\tau^{\prime })-\log\;p(x_{i};\hat{\theta }(\tau ),\tau )$, where $x_{i}$ is the pattern for the *i*th site. To test the null hypothesis that *τ* is correct, a one-sample *z*-test of $H_{0}\;:\;E[d_{i}]=0$ against $H_{A}\;:\;E[d_{i}]>0$ is used, giving *P*-value $p=P(Z>\bar{d})$, calculated with $Z∼N(0,s_{d}^{2})$, where $s_{d}^{2}$ denotes the sample variance of the $d_{i}$. Note that $n\bar{d}=l(\tau^{\prime })-l(\tau ),$ where l(τ) and l(τ′) give the maximized log likelihoods for the 2 trees. So, the KH test statistic is effectively the difference in maximized log likelihoods for the 2 trees. Kishino et al. (1990) present a variation that replaces the $N(0,s_{d}^{2})$ distribution with a bootstrap distribution. The bootstrap version, which bootstraps the $d_{i}$, is the one implemented by IQ-TREE; it gives very similar *P*-values to the normal-theory test.

Because the KH test is a *z*-test, the theory underlying the *z*-test implies that it would give a valid 2-tree test (with large sequence-length) if the $d_{i}$ were independent but this is not the case (Susko 2014). The estimation of *θ* induces dependencies between the $d_{i}$ and the distribution is more complex, involving the difference of 2 dependent random variables, each having a mixture of $\chi^{2}$ distribution. In simulations, the KH test has been found to be extremely conservative (probability of Type I error much less than *α*) in 2 tree settings.

When used in a confidence set construction procedure, $\tau^{\prime }$ is set to $\hat{\tau }$. Although the KH test is conservative for 2 fixed trees, because the ML tree, $\hat{\tau }$, is selected to make the log likelihood difference at least as large as it would be for any fixed $\tau^{\prime }$, selection bias gives a competing anti-conservative bias. Consequently, the expectation is that the probability of Type I error will increase or, equivalently, the coverage probability will decrease and may even fall below 1−α in the presence of severe selection bias.

#### The Chi-square Test

The chi-square test (Susko 2014) is a simple conservative test for 2 trees. Let *p* be the minimum number of edge-lengths that need to be set to 0 in order to make *τ* and $\tau^{\prime }$ the same; alternatively, it is the number of 0 edge-lengths in the strict consensus tree, $\tau_{0},$ of *τ* and $\tau^{\prime }$, and can be interpreted as the Robinson–Foulds (Robinson and Foulds 1981) distance between $\tau_{0}$ and $\tau^{\prime }$ or half the Robinson–Foulds distance between *τ* and $\tau^{\prime }$. The *P*-value for the chi-square test is calculated as the probability that a chi-square random variable with *p* degrees of freedom exceeds the observed 2-tree KH test statistic, $l(\tau^{\prime })-l(\tau )$. As with the KH test, when used in a confidence set construction procedure, $\tau^{\prime }$ is set to $\hat{\tau }$.

It was shown in Susko (2014) that, for 2 fixed trees, *τ* and $\tau^{\prime }$,


(1) $$P[2\{l(\tau^{\prime })-l(\tau )\}>y]\leq P[\chi_{p}^{2}>y].$$

It follows from this property that the chi-square test is also conservative although the simulations of Susko (2014) showed it to be much less conservative than the KH. As with the KH test however, when used to construct confidence sets, in which case $\tau^{\prime }=\hat{\tau }$, selection bias presents a competing anti-conservative bias. The coverage probability is expected to decrease as consequence of selection bias and can fall below 1−α for severe selection bias.

A refinement to the chi-square test can be used when p=1 or 2. We refer to it is the low degree of freedom correction. As discussed in Susko (2014),


(2) $$P[2\{l(\tau^{\prime })-l(\tau )\}>y]\approx \sum_{j=0}^{p}w_{j}P[\chi_{j}^{2}>y]$$

for some choice of $w_{j}\geq 0$ such that $\sum_{j=0}^{p}w_{j}=1$. Using that $P[\chi_{j}^{2}>y]\leq P[\chi_{p}^{2}>y]$ for j≤p gives (1) which is what the chi-square test is based on. The reason for using this latter inequality rather than (2) is that expressions for the $w_{j}$ are not generally known. In the case that p=1, however, $w_{0}=w_{1}=1/2$ (Ota et al. 2000) and in the case that p=2, $w_{1}=1/2$. So, one can still ensure that the chi-square test is conservative, yet obtain more power by calculating the *P*-value as


$$\begin{array}{rl} p=1: & P[\chi_{1}^{2}>2\{l(\tau^{\prime })-l(\tau )\}]/2\\ p=2: & P[\chi_{1}^{2}>2\{l(\tau^{\prime })-l(\tau )\}]/2+P[\chi_{2}^{2}>2\{l(\tau^{\prime })-l(\tau )\}]/2\\ p>2: & P[\chi_{p}^{2}>2\{l(\tau^{\prime })-l(\tau )\}]. \end{array}$$

We refer to this as the low degree of freedom correction of the chi-square test.

#### The SH Test

Shimodaira and Hasegawa (1999) introduced the SH test as a selection-bias modification of the KH test. Given a candidate set of *M* trees (in our case, all possible trees), the SH test samples sites with replacement. The log likelihood differences from these sites, are summed, which gives bootstrap log likelihoods, $l_{b}(j),$ for trees j=1,…,M and bootstrap samples b=1,…,B; larger is better and we used B=10,000 in all examples. In standard bootstrapping, bootstrap log likelihoods would be obtained by re-estimating parameters for each bootstrap sample and then summing the site log likelihoods for the bootstrap sample with these parameters. To save computational time, the SH test instead substitutes the estimated parameters from the original sample. The only thing that changes from bootstrap sample to bootstrap sample is which sites are being summed, not the log likelihood contributions from the sites, so this bootstrap variation is referred to as RELL resampling for “resampling estimated log likelihoods.”

The next step of the SH procedure is the reason that it is highly conservative. For each tree, *j*, its mean log likelihood over bootstrap samples is subtracted from each bootstrap likelihood, thus replacing $l_{b}(j),$ with a centered version, $l_{b}(j)-\sum_{b}l_{b}(j)/B$. The intent is to ensure that bootstrap generation is under a null hypothesis where the tree being tested is correct, in which case it should have maximal mean log likelihood. There are a number of ways to accomplish this goal and SH chooses the most conservative one, where the mean log likelihoods after subtraction are the same for all trees, so that every tree has a maximal expected log likelihood under bootstrap generation. This is consistent with the null hypothesis that all *M* trees are correct. Let $l_{b}(j),$ denote the centered log likelihoods. Then, a *P*-value is calculated as the proportion of *b* for which ${\text{max}}_{j}l_{b}(j)-l_{b}(\tau )$ is larger than the observed $l(\hat{\tau })-l(\tau )$. Topology *τ* is included in a (1−α)×100% confidence set if the *P*-value exceeds *α*. The maximum is used to mimic ML estimation for the bootstrapped samples and thus adjust for selection bias.

#### The AU Test

The AU test of Shimodaira (2002) is based on the bootstrap support, BP, of *τ*. As with the SH test, RELL resampling yields summed bootstrap log likelihoods, $l_{b}(j),$ for candidate trees, j=1,…,M; there is no centering. BP for *τ* is the proportion of *b* for which *τ* gave the largest $l_{b}(j)$. The AU test calculates ${\mathrm{BP}}_{r}$ for several fractions, *r*, of the original sequence length. For instance, with n=100 and r=1.1,  ${\mathrm{BP}}_{1.1}$ is calculated by repeatedly selecting 110 sites with replacement. Let Φ(z) denote the cumulative distribution function of the N(0,1) distribution. Having obtained ${\mathrm{BP}}_{r}$ for several choices of *r*, the procedure determines *c* and *d* satisfying that ${\mathrm{BP}}_{r}\approx 1-\Phi (d\sqrt{r}+c/\sqrt{r})$ using either a least squares approach or ML estimation, in the latter case treating the $nrBP_{r}$ as if they were independent binomial random variables. The CONSEL implementation of Shimodaira and Hasegawa (1999) uses ML estimation, whereas IQ-TREE uses the least-squares implementation. Either approach gives values of *d* and *c* that are substituted into 1−Φ(d−c) to give the AU *P*-value.

The theory that the AU test was based upon was developed by Efron and Tibshirani (1998) in a normal means setting. That theory used that 1−BP can be thought of as an approximate *P*-value in this setting. The hope was that results would extrapolate to 1−BP in phylogenetic settings but it was later shown that bootstrap support does not give an approximate *P*-value (Susko 2009). Consequently, the properties of the AU test are not really well understood.

A particular problem for the AU test arises when BP is very small. Since ${\mathrm{BP}}_{r}$ is BP but for a fraction *r* of the data that is close to the original sample size, one can expect that ${\mathrm{BP}}_{r}$ will tend to be small whenever BP is small. Recall that the AU *P*-value is effectively found by solving ${\mathrm{BP}}_{r}\approx 1-\Phi (d\sqrt{r}+c/\sqrt{r})$ for several choices of *r*. Equivalently, it solves $\Phi^{-1}(1-BP_{r})\approx d\sqrt{r}+c/\sqrt{r}$. Here $\Phi^{-1}(x)$ is the inverse cumulative distribution function of a N(0,1). Since, $\Phi^{-1}(x)$ blows up to ∞ when *x* approaches 1, then, when the ${\mathrm{BP}}_{r}$ are all small, small fluctuations in ${\mathrm{BP}}_{r}$ can cause enormous changes in $\Phi^{-1}(1-BP_{r})$ which lead to big changes in *d* and *c*, creating instability in the resulting 1−Φ(d−c) that gives the AU *P*-value. That this creates real problems was found in results where for many settings, large numbers of trees were included in AU-based confidence sets that had BP=0, a value of BP that clearly indicates a lack of support. The simple correction we use is to set AU *P*-values to 0 whenever BP = 0.

### Approximate Bonferroni Correction

The procedure that we refer to as a Bonferroni correction throughout can be applied to any test for 2 trees to correct for selection bias. These tests include the KH and chi-square tests as well as the other tests described in Susko (2014). We start by describing the procedure and then argue that, subject to some approximations to be discussed, the coverage of a (1−α)×100% confidence set using the procedure is expected to be at least 1−α.

The correction for tree *τ* depends upon the strict consensus tree, $\tau_{0}$, between *τ* and the ML tree $\hat{\tau }$. It also requires the subset, $A(\tau_{0})$, of trees in the candidate set of trees that are compatible with $\tau_{0}$. Our implementation obtained these quantities as follows. First, we determine the collection of splits of the taxa into 2 groups, A|B, present in any tree of the candidate set and give an integer label to every unique split found. Concurrently, we represent each topology as a vector of integers giving the indices of the internal splits present in that topology. The strict consensus tree, $\tau_{0},$ can then be represented as the set of integer indices present in both *τ* and $\hat{\tau }$, and $A(\tau_{0})$ is determined as the set of trees (represented as integer indices of splits) that include all of the integer indices present in $\tau_{0}$.

Let $p(\tau ,\tau^{\prime })$ denote the *P*-value coming from a 2-tree test of the null hypothesis that the true tree is *τ* against the alternative that it is $\tau^{\prime }$. For instance, for the chi-square test, this *P*-value would be calculated as $P[\chi_{p^{\prime }}^{2}>2\{l(\tau^{\prime })-l(\tau )\}]$ where $p^{\prime }$ is the number of zero-length edge-lengths in the strict consensus tree, $\tau_{0}$, of *τ* and $\tau^{\prime }$. Then, the Bonferroni-corrected *P*-value is calculated as


(3) $$1-{\left\{1-\min_{\tau^{\prime }\in A(\tau_{0})}p(\tau ,\tau^{\prime })\right\}}^{\left|A(\tau_{0})\right|}$$

where $\left|A(\tau_{0})\right|$ is the number of trees in $A(\tau_{0})$. Calculating the minimum can be costly as it requires multiple tests be applied for every choice of *τ*. An alternative version of the test, and the one we use most frequently in examples, replaces the minimum with $p(\tau ,\hat{\tau })$.

A less conservative Benjamini–Hochberg corrected *P*-value is given by $|A(\tau_{0})|\min_{k}p_{\left(k\right)}/k$ where the $p_{\left(k\right)}$ are obtained by ordering the $p(\tau ,\tau^{\prime })$ over $\tau^{\prime }\in A(\tau_{0})$ as $p_{\left(1\right)}<p_{\left(2\right)}<\cdots$. That approach, however, requires that for each *τ*, $p(\tau ,\tau^{\prime })$ be calculated over all $\tau^{\prime }\in A(\tau_{0})$, whereas the approach above requires only one *P*-value, $p(\tau ,\hat{\tau }),$ be calculated for each *τ*.

#### Coverage Properties

We now argue that the coverage of the procedure is expected to be at least 1−α. Due to the equivalence between hypothesis testing and confidence sets, it suffices to show that the Type I error rate is, up to approximation, at most *α* for the test that rejects when the Bonferroni-corrected *P*-value is less than *α*.

For a given tree *τ* of interest, we take our null hypothesis to be the strict consensus tree, $\tau_{0}$, of *τ* and $\hat{\tau }$. The rationale for this comes from usual statistical practice in calculating *P*-values when the distribution of the test statistic is not fully specified under the null hypothesis. First, one usually calculates *P*-values assuming parameters are on the boundary of the null and alternative hypotheses spaces. For instance, in testing $H_{0}\;:\;\theta \leq 0$ against $H_{A}\;:\;\theta >0$ for some parameter *θ*, *P*-values are usually calculated assuming θ=0. If they were calculated for some $\theta^{\prime }<0$, it would inflate Type I error rates whenever, in reality, the true *θ* was between $\theta^{\prime }$ and 0. Here, $\tau_{0}$ is on the boundary of tree space between the null (where *τ* is true) and the alternative. The other part of the rationale is to let the data be informative about what other parameters should be. For instance, for standard tests determined by comparing an estimate, $\hat{\theta }$, with a hypothesized value, a standard error for the estimator is required. If that standard error depends on additional unknown parameters, those parameters are estimated. Similarly, in considering very general hypotheses about means, Efron and Tibshirani (1998) argue that the appropriate null projects the unrestricted estimate onto the null hypothesis space. The null tree here, the strict consensus, $\tau_{0}$, of *τ* and $\hat{\tau }$, functions as the analog of a projection of $\hat{\tau }$ onto the null hypothesis space.

By consistency of tree estimation, if $\tau_{0}$ is the correct tree, then the set of alternatives that will arise will, with large probability, be in $A(\tau_{0}),$ the set of trees compatible with $\tau_{0}$. This is not guaranteed and depends on sequence lengths so it is our main approximation. Assuming this is the case, we can recast our hypotheses of interest as $H_{0}\;:$ the true tree is *τ* against $H_{A}\;:$ the tree is in $A(\tau_{0})$. This can be considered as a sequence of 2 tree hypotheses: for any $\tau \prime \in A(\tau_{0}),$  $H_{0}\;:$ the true tree is *τ* against $H_{A}\;:\;\tau^{\prime }$. Thus, we reject the null if any of the tests $H_{0}\;:\;\tau$ against $H_{A}\;:\;\tau^{\prime }$ rejects. Given a 2-tree testing procedure like the chi-square test or KH, we can obtain a *P*-value, $p(\tau ,\tau^{\prime })$ for this test. Because there are multiple tests, a correction is needed to ensure that the probability of rejection using the minimum *P*-value is small. The Bonferroni correction accomplishes this by using a corrected *P*-value, $\left|A(\tau_{0})|\min_{\tau^{\prime }\in A(\tau_{0})}p(\tau ,\tau^{\prime }\right)$. In applications, we replace this with the *P*-value (3) that one would obtain treating the tests as independent because that ensures a corrected *P*-value that is between 0 and 1. Having a *P*-value in (0,1) isn’t necessary but, as long as the Bonferroni *P*-value $|A(\tau_{0})|\min_{\tau^{\prime }\in A(\tau_{0})}p(\tau ,\tau^{\prime })\leq 0.1$, the values will be within 0.005 of each other. Differences between the *P*-values get larger when they are larger than 0.1 but such cases are not practically relevant to whether *τ* should be rejected or not.

Calculation of the minimum *P*-value requires that the test be applied for all $\tau^{\prime }\in A(\tau_{0})$. The alternative that we consider is to replace $\min_{\tau^{\prime }\in A(\tau_{0})}p(\tau ,\tau^{\prime })$ with $p(\tau ,\hat{\tau })$. Because $\hat{\tau }\in A(\tau_{0})$, by definition of a minimum, $p(\tau ,\hat{\tau })$ will be larger than the minimum *P*-value. So a test using $p(\tau ,\hat{\tau })$ in place of the minimum will reject less frequently. Since the Bonferroni correction ensures, up to approximation, that the test using the minimum *P*-value will have Type I error at most *α*, so will the test using $p(\tau ,\hat{\tau })$. Because $\hat{\tau }$ is the best supported tree, there is reason to expect that it will frequently give the smallest *P*-value. For the chi-square test, $\hat{\tau }$ gives the largest test statistic: $2[l(\hat{\tau })-l(\tau )]\geq 2[l(\tau^{\prime })-l(\tau )]$ for any $\tau^{\prime }\in A(\tau_{0})$ which suggests it will give the smallest *P*-value. However, it is possible that $p(\tau ,\tau^{\prime })$ will still be smaller than $p(\tau ,\hat{\tau })$ because the *P*-value is calculated as


(4) $$p(\tau ,\tau^{\prime })=P\{\chi_{p^{\prime }}^{2}>2[l(\tau^{\prime })-l(\tau )]\},$$

where $p^{\prime }$ is the number of edges that are set to 0 in the strict consensus of *τ* and $\tau^{\prime }$. By definition of $A(\tau_{0})$, $p^{\prime }\leq p$, where *p* is number of edges that are set to 0 in the strict consensus of *τ* and $\hat{\tau }$. So although the test statistic is larger using $\hat{\tau }$, the degrees of freedom are also larger.

The Benjamini–Hochberg correction applies to a more general setting and is less conservative than the Bonferroni correction. It was presented as a control on the false discovery rate for multiple tests and included a decision rule about which tests reject. Specifically, for an ordered set of *P*-values, $p_{\left(1\right)}<p_{\left(2\right)}<\cdots <p_{\left(M\right)},$ it rejects the *K* null hypotheses associated with $p_{\left(1\right)},\ldots ,p_{\left(K\right)}$, where *K* is determined as the largest *K* satisfying that $p_{\left(K\right)}\leq K\alpha /M$. The topology testing framework we are considering is a special case where all of the null hypotheses are true (that the true tree is *τ*), and we are only interested in whether any one of the tests rejects. Thus according to the Benjamini–Hochberg correction, we reject when $p_{\left(K\right)}\leq K\alpha /M$ for any *K* or equivalently when $M\min_{K}p_{\left(K\right)}/K\leq \alpha$. Thus, the smallest *α*-level that the test can reject at is $M\min_{K}p_{\left(K\right)}/K$ which can be used as a *P*-value. In Benjamini and Hochberg (1995), Type I error rates were shown to hold when the tests are independent. That is not the case here, as the same data are being used to obtain each $p(\tau ,\tau^{\prime })$. Benjamini and Yekutieli (2001) subsequently showed that the error rates still hold under a more general positive dependency condition. Although plausible, that condition is difficult to verify except in simple settings. Theorem 1.3 of Benjamini and Yekutieli (2001) presents a variation of the Benjamini–Hochberg procedure that applies without positive dependence but is more conservative than Bonferroni correction.

### Branch Support Values and Confidence Sets

Confidence sets of topologies can be used to define branch support values and provide a way of connecting the Bonferroni correction to the aLRT test of Anisimova and Gascuel (2006). The aLRT support value for a split *s* has an interpretation as 1−p where *p* is a *P*-value of the null hypothesis of $H_{0}$ : *s* is not present against $H_{A}$ : *s* is present. Bootstrap support (BP) was originally presented as having this interpretation (Felsenstein and Kishino 1993; Efron et al. 1996). It was subsequently shown (Susko 2009) that it is conservative: BP>0.95 is expected to happen less than 5% of the time when *s* actually is present. Those results, however, were for a fixed split, *s*. There can be a selection bias here too. BP for the splits in the ML tree (which is selected based on the data) tends to be larger than for any particular fixed tree. The aLRT is intended for the ML tree whereas results for BP apply to fixed trees.

To obtain a support value from a confidence set procedure, first consider the test that rejects when every tree in the (1−α)×100% confidence set has the split *s*. Consider any tree *τ* satisfying the null hypothesis that the split *s* is not present. When the test rejects, it implies *τ* is not in the confidence set. Since P(A)≤P(B) whenever *A* implies *B*,


$$P_{\tau }[\text{reject }H_{0}]\leq P_{\tau }[\tau \text{ not in confidence set}]=1-P_{\tau }[\tau \text{ in confidence set}]\leq 1-(1-\alpha )=\alpha .$$

Since this holds for any *τ* satisfying $H_{0}$, the test is an *α*-level test. Because of the first inequality, it is a conservative test even if the confidence set construction procedure had coverage probability close to the nominal level of (1−α).

One can construct a *P*-value and hence a support value using that *P*-values are generally defined as the smallest *α*-level at which the test could reject. Let p(τ) denote the *p*-value that was used to determine whether *τ* is in the confidence set and let $T_{s}$ denote the set of trees with the split. The test for the split *s* rejects iff all $\tau \notin T_{s}$ have p(τ)≤α or, equivalently, if ${\text{max}}_{\tau \notin T_{s}}p(\tau )\leq \alpha$. Thus, the smallest *α* at which the test rejects is ${\text{max}}_{\tau \notin T_{s}}p(\tau )$ and the corresponding support value is $1-{\text{max}}_{\tau \notin T_{s}}p(\tau )$.

#### aLRT Support Values

The aLRT support values are for the ML tree. It assumes *s* may or may not be present but that the rest of the structure in the tree is present. This implies that the tree can be represented as AB|CD, AC|BD, or AD|BC where *A*, *B*, *C*, and *D* are subtrees that are present in the ML tree. Assume, without loss of generality, that the split in the ML tree being tested is AB|CD and let $l_{1}$ denote its log likelihood. Let $l_{2}$ denote next best log likelihood among those of AC|BD and AD|BC. Then, the aLRT support value of Anisimova and Gascuel (2006) is $1-3[1-F^{*}(2\{l_{1}-l_{2}\})]$, where $F^{*}(x)=1/2+P[\chi_{1}^{2}\leq x]/2$. Similarly as here, Anisimova and Gascuel (2006) consider an alternative support value that arises by treating the tests as independent and is very close to the Bonferroni value when support is large but has the advantage of always being in [0,1]. Referred to as the cubic correction, it is $F^{*}(2\{l_{1}-l_{2}\}{)}^{3}$. We use this latter expression throughout.

The 4-taxon case provides a means of relating aLRT values to support values for the ML split obtained using the chi-square and Bonferroni tests presented earlier. In the 4-taxon case, *A*, *B*, *C* and *D* represent taxa. We assume a chi-square test using a low degree of freedom correction. Then the chi-square test assigns *P*-values $1-F^{*}(2\{l_{1}-l_{2}\})$ and $1-F^{*}(2\{l_{1}-l_{3}\})$ to the topologies other than the ML topology. Since $l_{2}\geq l_{3}$, the maximum *P*-value over trees not having the ML split is $1-F^{*}(2\{l_{1}-l_{2}\}),$ resulting in support value $F^{*}(2\{l_{1}-l_{2}\})$ that will always be larger than aLRT. Similarly, the maximum Bonferroni *P*-value is calculated with $l_{2}$. From (3) we obtain


$$1-{\left\{1-\min_{\tau^{\prime }\in \{1,2\}}[1-F^{*}(2\{l_{1}-l_{\tau^{\prime }}\})]\right\}}^{2}=1-\{1-[1-F^{*}(2\{l_{1}-l_{2}\})]{\}}^{2}=1-F^{*}(2\{l_{1}-l_{2}\}{)}^{2}.$$

Thus, the Bonferroni support value $F^{*}(2\{l_{1}-l_{2}\}{)}^{2}$ is always larger than the aLRT support value, $F^{*}(2\{l_{1}-l_{2}\}{)}^{3}$. This is because support values from confidence sets are for fixed splits whereas aLRT adjusts for selection of the ML tree.

The aLRT support values assume that the only structure in the tree that is not present is the split of interest. When neighboring edges are not small, this assumption is not likely to be too problematic. Because the ML tree is AB|CD, it is expected in this case that the only well-supported trees will be AB|CD, AC|BD, or AD|BC. When some neighboring edges are small, the ML tree was effectively a maximum over more than 3 trees and the aLRT support value under-corrects.

#### aLRT Confidence Sets

Intuitively, it makes sense that one could construct a confidence set from aLRT support values as the set of trees that have no splits that conflict with supported splits in the ML tree. The true tree is then in the set if all of the incorrect splits in the ML tree are not supported. If the conditional probability that a split is supported, given that it is incorrect, is at most $\alpha^{\prime }$, then the joint probability that a randomly selected split is incorrect and supported is at most $\alpha^{\prime }$ too. Let *m* be the number of taxa. Then, since there are m−3 nontrivial splits in the true tree, the probability that at least one of them is incorrect and supported is at most $(m-3)\alpha^{\prime }$. Thus the probability that the true tree is in the confidence set is at least $1-(m-3)\alpha^{\prime }$.

For the aLRT test, given an incorrect split, the probability of support exceeding $1-\alpha^{\prime }$ is at most $\alpha^{\prime }$. Consider the confidence set defined as the set of trees that have no splits that conflict with supported splits in the ML tree, where now a supported split is one whose aLRT support value exceeds 1−α/(m−3). Then the probability that the true tree is in the confidence set is at least 1−(m−3)α/(m−3)=1−α. We refer to the confidence set constructed in this manner as the aLRTc set. What we see here is that in converting from support for splits to support for trees, we need to adjust for the multiplicity of tests over splits. Since, equivalently, the aLRTc supports a split when 1−(m−3)(1−aLRT)≥1−α, we refer to 100[1−(m−3)(1−aLRT)]% as the aLRTc support value for a split.

The *P*-value for the equivalent test of the null hypothesis that the true tree is τ, is the smallest *α*-level for which the test rejects. The test rejects at the *α*-level if *τ* is not in the (1−α)×100% confidence set. Let M(τ) denote the maximum support over splits in the ML tree that are incompatible with *τ*. For aLRT, *τ* is not in the confidence set if M(τ)≥(1−α). So the smallest *α*-level is obtained by setting M(τ)=1−α or giving *P*-value 1−M(τ). For the aLRTc version, we set M(τ)=1−α/(m−3) which gives corrected *P*-value (m−3){1−M(τ)}.

There is a caveat in all of the discussion above: it was assumed that the aLRT gives conditional probability of any particular incorrect split having support exceeding $1-\alpha^{\prime }$ that is at most $\alpha^{\prime }$. That result was argued for in Anisimova and Gascuel (2006) assuming that there is only one zero-length edge. It was not calculated for a fixed tree that might have multiple zero-length edges, which could increase the chance that an incorrect split is supported.

## Data Availability

All data sets used in this paper are available upon request.
